# Defect-Rich Gas–Solution Photocatalytic Systems for Nitrogen Reduction Reactions: Enabling Energy and Carbon Reductions

**DOI:** 10.1021/acsomega.5c07318

**Published:** 2025-11-25

**Authors:** Shih-Mao Peng, Muhammad Saukani, Jen-Chang Yang, Tsung-Rong Kuo

**Affiliations:** † Graduate Institute of Biomedical Materials & Tissue Engineering, 38032 Taipei Medical University, New Taipei City 23564, Taiwan; ‡ Department of Mechanical Engineering, Faculty of Engineering, Universitas Islam Kalimantan MAB, Banjarmasin 70124, Kalimantan Selatan, Indonesia; § Graduate Institute of Nanomedicine and Medical Engineering, College of Biomedical Engineering, Taipei Medical University, Taipei 23564, Taiwan; ∥ International Ph.D. Program in Biomedical Engineering, College of Biomedical Engineering, Taipei Medical University, New Taipei City 23564, Taiwan

## Abstract

The efficient and sustainable production of ammonia is pivotal for global food security and energy sustainability. In this study, we developed a novel gas–solution (G–S) photocatalytic nitrogen reduction reaction (PNRR) system utilizing molybdenum oxide deposited onto carbon fiber paper (MoO_3_@CFP) and a low-cost 24 W plant lamp as the illumination source. The G–S system eliminates the need for nitrogen gas bubbling by relying on ambient air as the nitrogen source and was demonstrated to be a simplified and scalable approach to ammonia production. The amorphous structure of MoO_3_@CFP provides abundant active sites and defect centers, enabling effective nitrogen activation and reduction. Under optimized conditions (current = 0.25 A, deposition time = 600 s, stirred), the system achieved a mass-normalized ammonia production rate of 15.144 mmol·g^–1^·h^–1^ and sustained performance over five consecutive cycles (1 h per cycle). Material characterization confirmed the structural integrity and compositional stability of the catalyst after repeated use. A working mechanism is proposed in which Mo–O–Mo linkages and defect sites facilitate electron transfer and nitrogen activation. Overall, this study introduces a cost-effective route to photocatalytic ammonia synthesis using ambient air in a G–S configuration.

## Graphical abstract

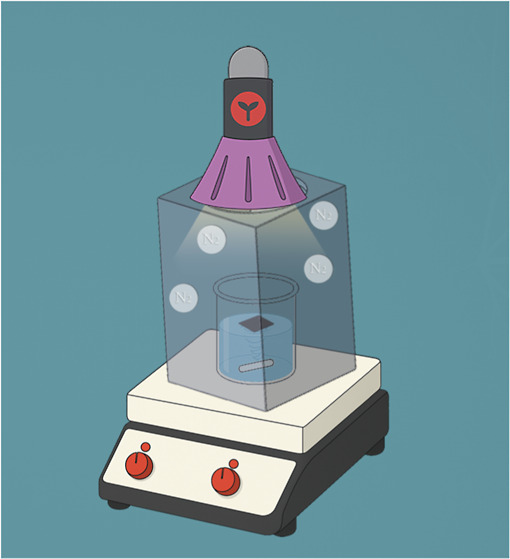

## 1 Introduction

Ammonia (NH_3_) has garnered significant attention for its broad applications as a green fertilizer, carbon-free fuel, and efficient energy carrier, offering practical solutions to carbon neutrality and the intermittency of renewable energy. Its high energy density, carbon-free fuel, and established global supply chain make NH_3_ a leading candidate for zero-carbon fuels, particularly in heavy transportation, fuel cells, gas turbines, and marine engines.^1,2^ However, conventional ammonia synthesis via the Haber–Bosch process remains energy-intensive, as it requires high temperatures and pressures, which raises environmental and sustainability concerns.^3,4^ In contrast, renewable-energy-driven catalytic approaches for “green ammonia” production provide sustainable alternatives with immense potential for large-scale fossil fuel displacement.^5–7^ As energy systems worldwide advance toward decarbonization, the versatility and scalability of ammonia reinforce its pivotal role in shaping the future of sustainable energy.^8,9^

Catalytic ammonia synthesis methods include photocatalysis,^10,11^ electrocatalysis,^12,13^ and biocatalysis.^14–16^ Among these, photocatalysis, inspired by natural photosynthesis, directly converts solar energy into chemical energy and has emerged as a promising strategy for photoreactions. This sunlight-driven approach not only eliminates the energy input required for electrocatalysis but also enables sustainable NH_3_ production using natural energy sources. Conventional photocatalysts, including metals (noble metals,^17–19^ main group metals,^20–24^ and transition metals^25–30^ ), sulfides,^31–35^ and carbon-based structures,^36–48^ have been extensively studied for this reaction. However, their catalytic efficiency is often constrained by an insufficient number of active sites for binding and activating the strong N≡N triple bond (941 kJ mol^–1^). Single-atom catalysts (SACs) offer a compelling alternative by increasing the availability of active sites and maximizing atomic efficiency. Despite these advantages, achieving precise control over the local chemical environment surrounding active sites remains a significant challenge.

Ambient-condition photocatalytic nitrogen reduction (PNRR) has advanced across defined catalyst classes: noble-metal,^49^ transition-metal,^50–53^ g-C_3_N_4_.^54,55^ Across these systems, common strategies include defect engineering (oxygen vacancies/under-coordination), type-II and Z-scheme heterojunctions, plasmonic/metallization to promote charge separation, and carbon scaffolds to facilitate electron extraction—collectively underscoring that interfacial charge transfer and mass transport at solid–liquid–gas boundaries cogovern performance. In the field of photocatalytic nitrogen reduction reaction (PNRR) research, many catalytic materials have demonstrated exceptional performances but are typically employed in gas–liquid interface systems (GISs), where nitrogen gas interacts with water to facilitate reactions at the gas–solid interface.^56^ This approach relies on the interplay among nitrogen gas, photons, photoelectrons, and protons. However, GISs face several critical challenges: (I) the solubility of nitrogen in water is extremely low; (II) nitrogen transfer at the interface is sluggish; and (III) direct contact between photocatalysts and water introduces excess protons, which overwhelm the catalytic sites and hinder effective nitrogen activation. Advanced porous framework photocatalysts have been developed to address these issues, yet water infiltration into the porous channels remains a major obstacle. Recent studies suggested that solvent-in-gas (SIG) systems, where photocatalysts operate in nitrogen-rich environments with uniformly dispersed proton sources, offer a promising alternative. These systems significantly enhance the catalytic efficiency by resolving challenges related to nitrogen solubility and diffusivity, allowing nitrogen molecules with high diffusion coefficients to dominate the reaction environment.

In this study, we fabricate amorphous, defect-engineered molybdenum oxide deposited onto carbon fiber paper (MoO_3_@CFP) and deploy it in a gas–solution (G–S) platform for photocatalytic nitrogen reduction. Our approach uses precursor-concentration control and applied-current tuning during electrodeposition to regulate catalytically relevant defect motifs while preserving the amorphous structure. We establish a bubbling-free G–S reactor using ambient air as the N_2_ source, and systematically evaluate operating variables (current, reaction time, hydrodynamics, and sacrificial agents) alongside comparative solution, gas, and G–S system configurations and cycling tests. Coupling performance measurements with complementary characterization (e.g., XRD, Raman, XPS, SEM, electrochemical impedance spectroscopy (EIS)/diffuse reflectance spectroscopy (DRS)), we correlate structural features with activity and propose a working mechanism in which Mo–O–Mo linkages and defect-associated sites enable charge transfer and N_2_ activation. This framework aims to provide a practical, low-cost route to G–S system PNRR and to clarify design principles for future defect-engineered catalysts.

## 2 Materials

### 2.1 Chemicals

Molybdic acid (H_2_MoO_4_; ACS 85% minimum) was procured from ChemScene. Trisodium citrate (C_6_H_5_Na_3_O_7_; ACS grade) was procured from Fisher. Carbon fiber paper (CFP) was procured from CeTech. Sodium sulfate (Na_2_SO_4_; ACS 99%) was procured from Sigma-Aldrich. Sodium hypochlorite (NaClO; 11–15% active Cl basis) was procured from Scharlau. Sodium pentacyanonitrosylferrate­(III) dihydrate (Na_2_[Fe­(CN)_5_NO]·2H_2_O; ACS 99+%) was procured from Alfa Aesar. Phenol (C_6_H_5_OH; ACS grade) was procured from Scharlau. Sodium hydroxide (NaOH; ACS grade) was procured from PanReac AppliChem. Distilled water and absolute ethanol were used throughout the experiments.

### 2.2 Characterization

The morphologies of the samples were examined by scanning electron microscopy (SEM) and energy-dispersive X-ray (EDX) mapping using a Hitachi SU3500 microscope. A structural analysis was performed via X-ray diffraction (XRD) using a D2 PHASER (Bruker). Raman spectra were recorded using a UniDRON laser spectroscopy confocal micro Raman spectroscopy system. Electrochemical measurements, including electrochemical impedance spectroscopy (EIS), were conducted with a Metrohm Autolab PGSTAT204 (Metrohm Autolab). An X-ray photoelectron spectroscopy (XPS) analysis was performed using an ULVAC-PHI Quantes instrument. Additional electrochemical characterization was performed with a CHI627E workstation.

### 2.3 Synthesis of the Cathode of MoO_3_@CFP and the Anode of MoO_*x*_@CFP

The MoO_3_@CFP and MoO_*x*_@CFP were synthesized through a controlled electrochemical deposition approach. Here the stated current (0.125–0.25 A) and time (300–600 s) refer exclusively to electrodeposition conditions that define film properties; no external current is applied during PNRR tests. CFP was prepared by sectioning it into 1 × 5 cm strips, with a defined 1 × 2 cm region immersed in an electrolyte solution. The electrolyte consisted of 1 M trisodium citrate and 1 M H_2_MoO_4_, and the bath was magnetically stirred at 1000 rpm throughout deposition to maintain homogeneous mass transport. A two-electrode electrochemical configuration was employed, with the CFP serving as the working electrode. Deposition was achieved by applying a constant current of 0.25 A for 600 s, ensuring uniform molybdenum oxide deposition onto the CFP surface. Postdeposition, samples were meticulously rinsed with deionized water to remove residual electrolytes and dried at 100 °C for 15 min to stabilize the formed structure. This process yielded the MoO_3_@CFP and MoO_*x*_@CFP.

### 2.4 Photocatalytic Nitrogen Reduction Reaction (PNRR)

The PNRR was performed in a controlled setup under visible light irradiation as shown in Figure S1. The experiment commenced with 100 mL of ultrapure water in a reaction vessel. The 2 × 1 cm^2^ catalytic material was introduced and carefully positioned to float on the liquid surface, maximizing its exposure to the light source. To suppress ambient contamination and standardize the headspace, the reaction was conducted inside a light-tight dark box (internal dimensions 5 × 5 × 14 cm; height 14 cm) with a single circular aperture (*Ø* 8 cm) on the top panel. Before each run, all internal surfaces, glassware, and fixtures were sequentially cleaned with deionized water, ethanol, and acetone, and then air-dried. After the reactor and catalyst were loaded, the enclosure was briefly purged with nitrogen to displace residual laboratory air; the plant lamp was subsequently seated directly over the top aperture to form a near-sealed configuration while maintaining a nonbubbling, diffusion-controlled headspace. To monitor the progress of the reaction, 1 mL aliquots were sampled from the solution at 12 min intervals. These aliquots were analyzed to determine the concentrations of NH_3_/NH_4_^+^, providing real-time data on the efficiency of the PNRR. The experimental setup was carefully controlled to ensure accurate quantification and reproducibility, enabling an in-depth evaluation of the photocatalyst’s performance under visible light conditions. The light source utilized in this study was a 24 W plant lamp with a spectral range spanning ca. 380–780 nm, encompassing ultraviolet (UV), visible (vis), and near-infrared (IR) regions. The light featured a peak wavelength of 452.1 nm and a main wavelength of 566.8 nm, ensuring efficient excitation of the photocatalyst. The photosynthetic photon flux density (PPFD) at 100 cm was 150 μmol·m^–2^·s^–1^, providing sufficient photon availability to drive photocatalytic reactions. The spectral characteristics closely mimicked natural sunlight with a correlated color temperature of 5119 K and a high color rendering index (CRI Ra = 97.0). These parameters made the light source cost-effective and well-suited for PNRRs, offering a stable and uniform light distribution across the reaction system. Experiments were performed in a light-tight enclosure under ambient air; unless otherwise specified, temperature was room temperature.

### 2.5 Detection of Ammonia (NH_3_)

Ammonia concentrations were quantified using the indophenol blue method combined with UV–vis spectrophotometry. A 1 mL aliquot from the reaction vessel was mixed with 100 μL of an oxidizing solution containing NaClO in 1 M NaOH (pH 11–15). Subsequently, 100 μL of 0.5 M phenol and 50 μL of a 0.002 M sodium pentacyanonitrosylferrate­(III) dihydrate (Na_2_[Fe­(CN)_5_NO]·2H_2_O; ACS 99+%) solution were sequentially added. The mixture was gently agitated for 30 s and left in the dark for 30 min to develop the characteristic indophenol blue color. Absorbance measurements were performed at a wavelength of 650 nm. A calibration curve was established using NH_4_^+^ standard solutions prepared with NH_4_Cl at concentrations ranging 0.1–1 μg·mL^–1^. The calibration curve exhibited a linear relationship between the absorbance and NH_3_ concentration, defined by the equation *y* = 0.07022*x* + 0.00381 with an *R* ^2^ value of 0.98983. Quantification was performed by external calibration within this range; samples outside the range were diluted accordingly. Procedural and reagent blanks were processed alongside samples and their absorbance contributions subtracted. The calibration exhibited good linearity (*R* ^2^ = 0.98983) within 0.1–1 μg·mL^–1^.

### 2.6 Electrochemical Analysis

The electrochemical performance of MoO_3_@CFP was evaluated using a standard three-electrode system (CHI627E), where a Pt wire served as the counter electrode and Ag/AgCl as the reference electrode. The transient light current response was measured under illumination from a 24 W plant lamp in a 0.5 M Na_2_SO_4_ electrolyte solution. Electrochemical impedance spectroscopic (EIS) measurements were further conducted using a Metrohm Autolab PGSTAT204 under identical conditions to assess the charge transfer properties and impedance behavior of MoO_3_@CFP.

## 3 Results and Discussion

### 3.1 Characterization of the Cathode of MoO_3_@CFP

The XRD pattern (Figure 1a) of MoO_3_@CFP shows only the (002) reflection of graphitic carbon from the substrate, with no reflections of crystalline molybdenum oxides, indicating an amorphous MoO_*x*_ phase. Raman spectrum of MoO_3_@CFP (Figure 1b) displays broad Mo–O–Mo-related bands spanning ∼240–300 and ∼520–850 cm^–1^, consistent with an amorphous MoO_*x*_ network. A contribution ∼820–830 cm^–1^ is attributable to bridging Mo–O–Mo stretching, in line with the FTIR peaks at 723 and 848 cm^–1^ (Figure 1d). Features around ∼750–780 cm^–1^ arise from symmetric stretching within the Mo–O–Mo framework. The terminal Mo=O mode expected near ∼950–1000 cm^–1^ is weak/broad and not clearly resolved by Raman spectra and is instead corroborated by FTIR peak of MoO_3_@CFP at 963 cm^–1^ (Figure 1d). The carbon D (∼1350 cm^–1^) and G (∼1600 cm^–1^) bands of CFP remain visible, indicating preservation of the CFP framework. XPS spectrum of MoO_3_@CFP (Figure 1c) shows Mo 3d_5/2_ and Mo 3d_3/2_ components at 232.4 and 235.6 eV, characteristic of Mo­(VI) in molybdenum oxides. FTIR spectrum of MoO_3_@CFP (Figure 1d) exhibits a band at 963 cm^–1^ attributable to terminal Mo=O stretching and bands at 723 and 848 cm^–1^ arising from bridging Mo–O–Mo vibrations, corroborating the presence of molybdenum oxide. SEM imaging (Figure 2a,b) provided further insights into the microstructure of MoO_3_@CFP, showing a uniformly coated carbon fiber network with visible cracks attributed to the deposition process. An elemental composition analysis using EDX mapping (Figure 2c) revealed the presence of carbon, molybdenum, and oxygen, at respective area-averaged compositions from multiple, nonoverlapping fields were 44.53 wt % C, 21.95 wt % Mo, and 33.49 wt % O; 61.48 at. % C, 3.80 at. % Mo, and 34.72 at. % O. Given matrix effects on the carbon substrate, these values are semiquantitative; uniformity is assessed from the elemental maps (Figure 2d–f), which show homogeneous Mo and O along the CFP fibers. Oxidation states/bonding environments are addressed by XPS (Mo 3d, O 1s).

**Figure 1:**
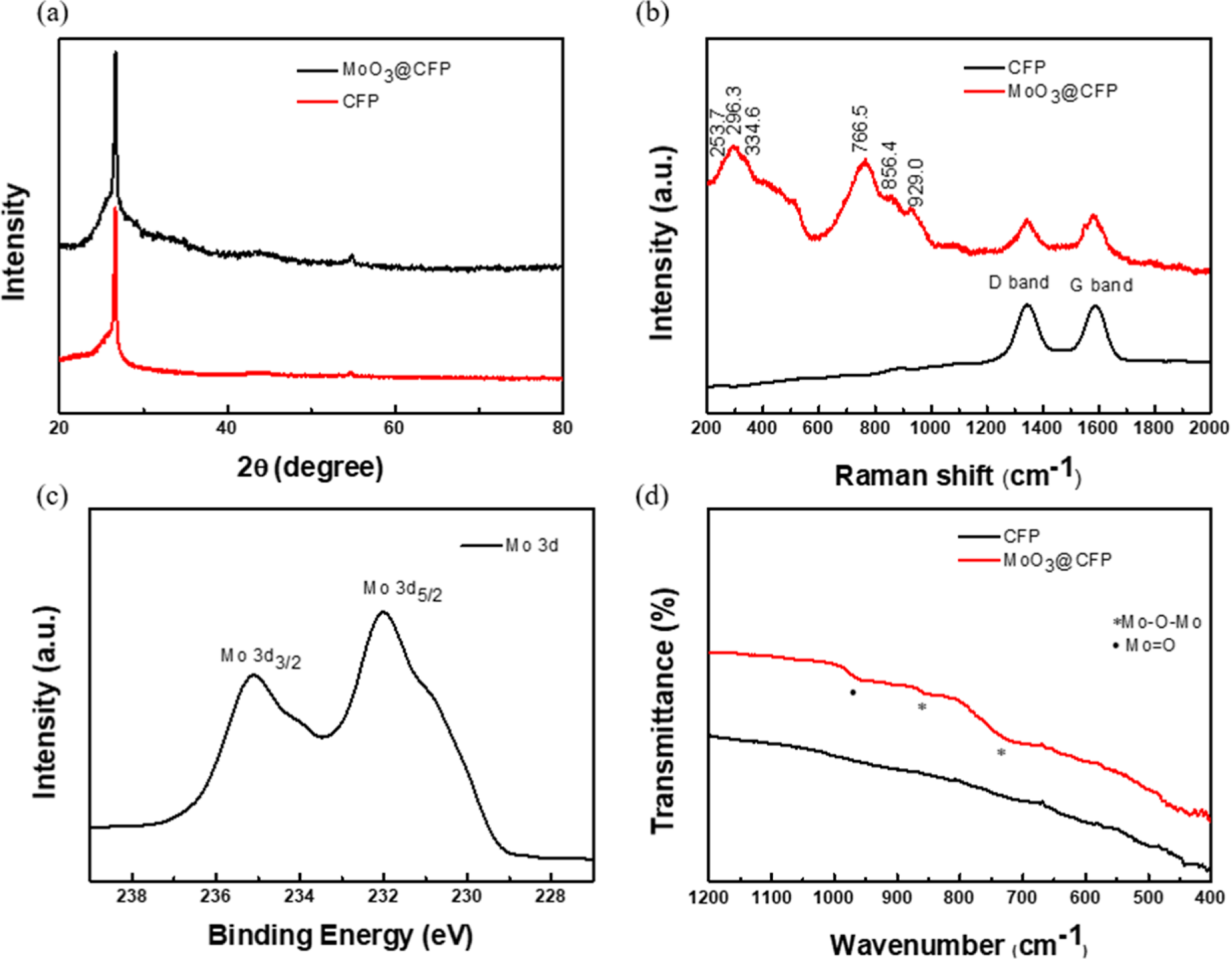
Characterization data for MoO_3_@CFP (a) XRD (b) Raman (c) XPS and (d) FTIR.

**Figure 2:**
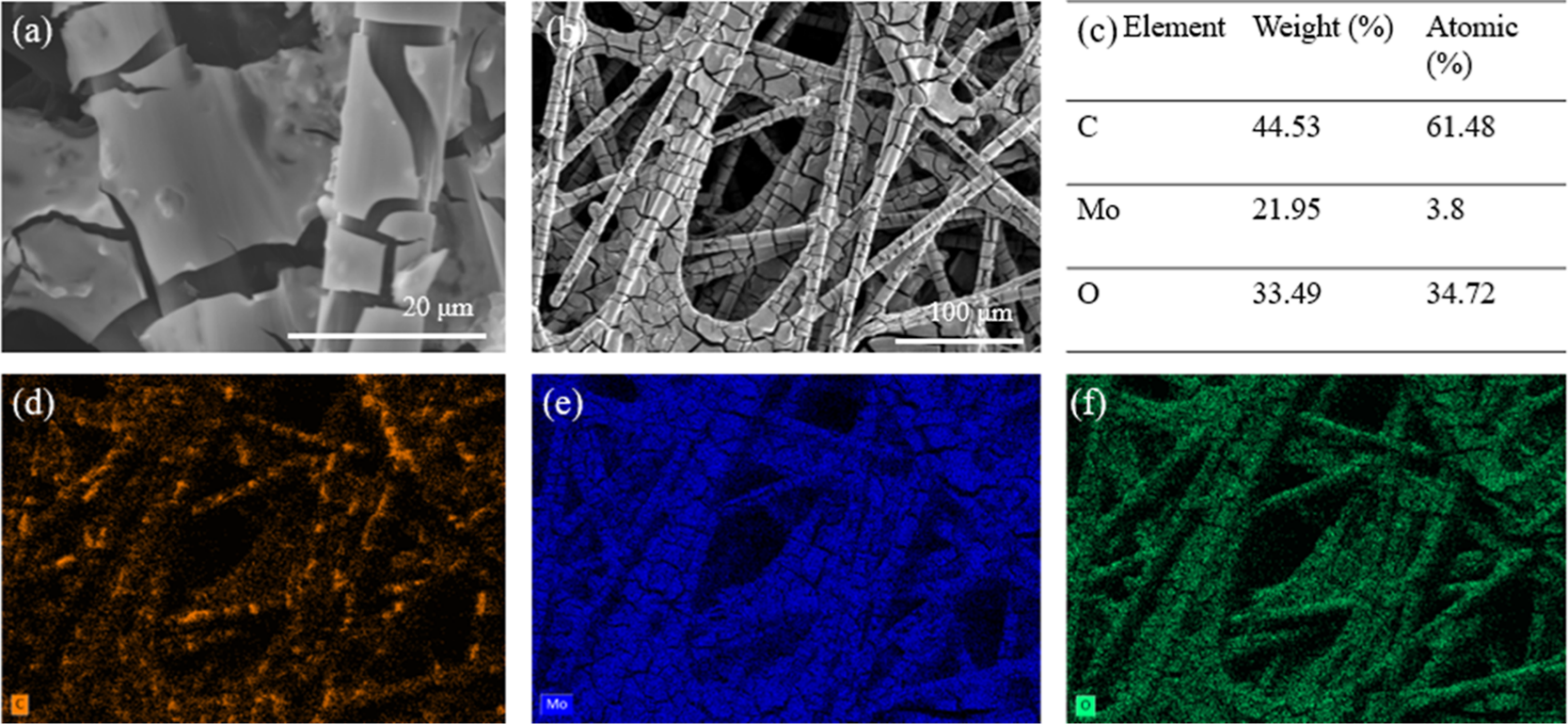
(a,b) SEM images of the MoO_3_@CFP cathode at different scales; (c) EDX analysis showing elemental composition; and EDX element mapping of (d) C, (e) Mo, and (f) O.

### 3.2 Different Reaction Environment Photocatalytic Activities

The PNRR performance of MoO_3_@CFP and MoO_*x*_@CFP was comprehensively evaluated under three distinct environments: solution, gas, and G–S Systems. The UV–vis absorption spectra (Figures 3 and S2) served as a direct indicator of ammonia production, with the absorption peak intensity at 650 nm which strongly correlated with the generated NH_3_ concentration. Among the tested environments, the G–S system demonstrated a significant enhancement in catalytic performance, achieving superior absorbance values compared to the solution and gas systems, particularly over prolonged reaction durations (e.g., 3 h). Figure 3a–c illustrate the experimental configurations, where the G–S system effectively bridged the benefits of a high nitrogen concentration in the gas phase and efficient proton transfer from the liquid phase. This unique synergy overcame the intrinsic limitations of the conventional single-phase systems. For example, the solution system was constrained by the inherently low solubility of nitrogen in water, leading to a reduced reaction rate. Similarly, the gas system, while offering high nitrogen availability, suffered from poor mass transfer and reactant delivery to the catalytic interface. The time-dependent absorbance trends further highlighted the advantages of the G–S system. Compare each reaction environment, the solution system (Figure 3d) exhibited a plateau in absorbance, indicative of nitrogen depletion or transport limitations, while the gas system (Figure 3e) demonstrated only marginal improvements over time due to mass transfer resistance. Figure 3f shows a steady increase in absorbance intensity over 1, 2, and 3 h, suggesting enhanced reaction kinetics and efficient utilization of reactants in this hybrid environment. Furthermore, Figure S3 provides additional insights into the impacts of operational parameters such as the current density and reaction duration on the PNRR performance. At a higher current of 0.25 A, the system achieved optimal ammonia production, as reflected by the elevated absorbance intensity. Notably, quantitatively, under the G–S configuration the mass-normalized rate reaches 15.144 mmol·g^–1^·h^–1^ (Table 2), exceeding the solution and gas cases under otherwise identical conditions. The inclusion of magnetic stirring further enhanced the reaction efficiency, underscoring the importance of optimizing reaction conditions to maximize the catalytic activity. The side-by-side comparison across solution, gas, and G–S with an identical catalyst/light source isolates the transport geometry; the divergent time-dependent responses thus serve as empirical evidence for G–S synergy without recourse to external *k*-measurements.

**Figure 3:**
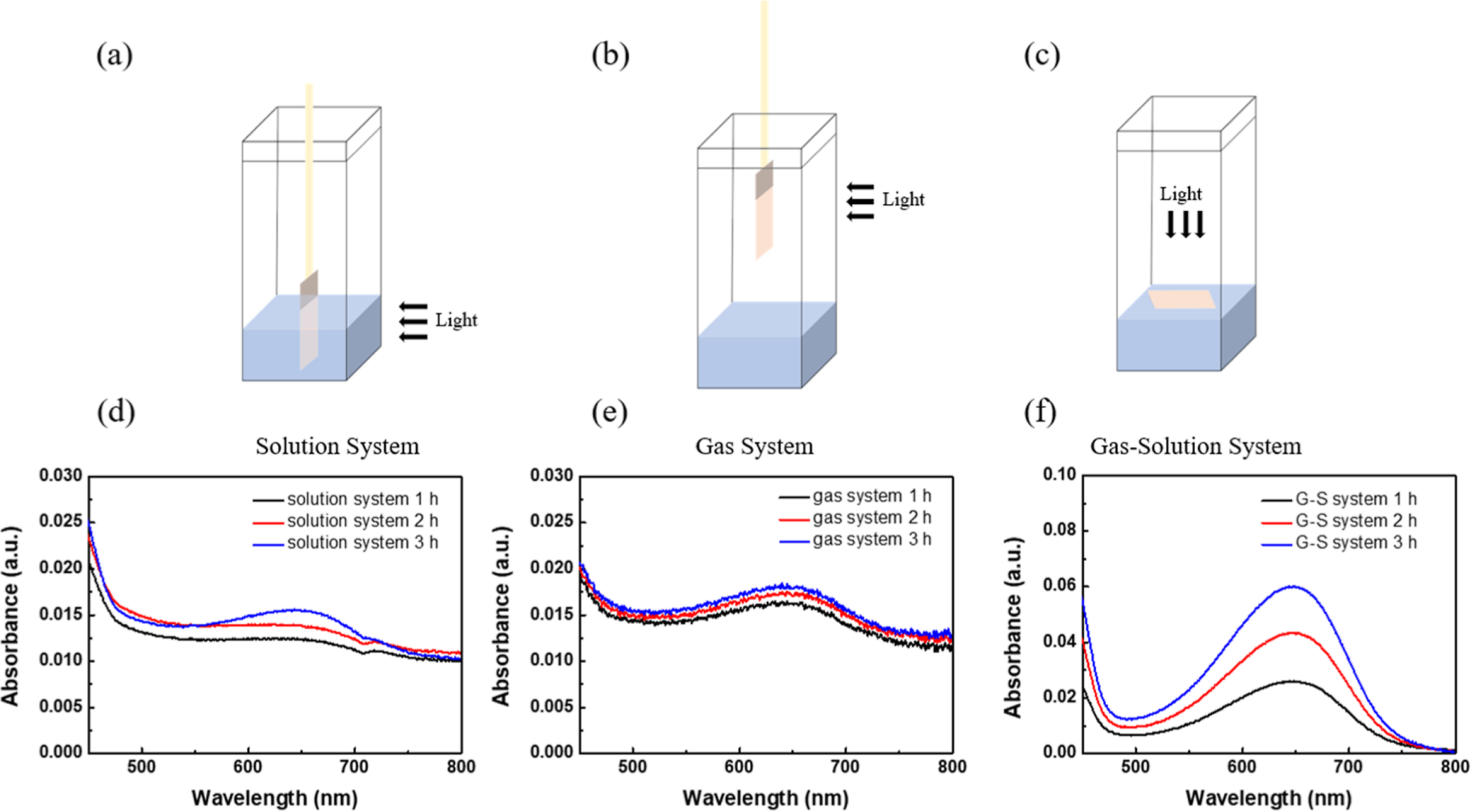
Illustration of the PNRR reaction setup: (a) solution system, (b) gas system, and (c) G–S system. Panels (d–f) depict the UV–vis absorption spectra of ammonia production in these systems at different reaction times (1, 2, and 3 h).

### 3.3 Comparing PNRR Effects of Different Electrode Cathodes of MoO_3_@CFP and Anodes of MoO_*x*_@CFP

The PNRR performances of MoO_3_@CFP and MoO_*x*_@CFP in Figure 4 are presented as UV–vis absorption spectra, providing a detailed comparison of the two configurations under various electrochemical deposition durations during material preparation. Figure 4a,b respectively illustrate the absorption spectra of the cathode and anode configurations, showing the time-dependent response of ammonia production over 1, 2, and 3 h. For the cathode (Figure 4a), the absorbance intensity exhibited a steady increase across all reaction durations, reflecting efficient nitrogen activation and reduction. In contrast, the anode (Figure 4b) displayed a slower increase in absorbance, indicating a lower overall catalytic efficiency. Figure 4c,d further explore the effect of electrochemical deposition times during material preparation (600 vs 300 s) on the catalytic performance. The cathode prepared with a shorter deposition time (300 s) still demonstrated measurable activity, as shown in Figure 4c, although with slightly reduced absorbance compared to the 600 s sample. For the anode (Figure 4d), the shorter deposition time significantly reduced the overall performance, suggesting that the electrode preparation process critically impacts the density of active sites and electron transfer pathways. The quantitative data presented in Table 1 complement these spectral findings. As shown in Table S1, after subtracting the weight of the CFP substrate, the average weights of MoO_3_@CFP and MoO_*x*_@CFP were respectively calculated to be 0.8 and 2.7 mg. These values highlight significant differences in material loading, which could partially explain the variation in catalytic efficiency between the two configurations. Given the fibrous CFP scaffold and thin-film architecture, conventional BET is dominated by macroporosity and is not diagnostic for the effective film surface. We therefore compare performance on a mass-normalized basis and assess uniformity from SEM/EDX mapping (Table S1/Figure 2). When normalized to the catalyst mass, the cathode achieved an exceptional ammonia production rate of 15.144 mmol·g^–1^·h^–1^ under the G–S system, further validating its superior efficiency. The cathode’s robust electron donor properties and abundant surface defect sites contributed to its enhanced catalytic activity. In contrast, the anode’s performance was hindered by oxidative side reactions and less-efficient utilization of active sites, which were exacerbated when the electrochemical deposition time was reduced.

**Figure 4:**
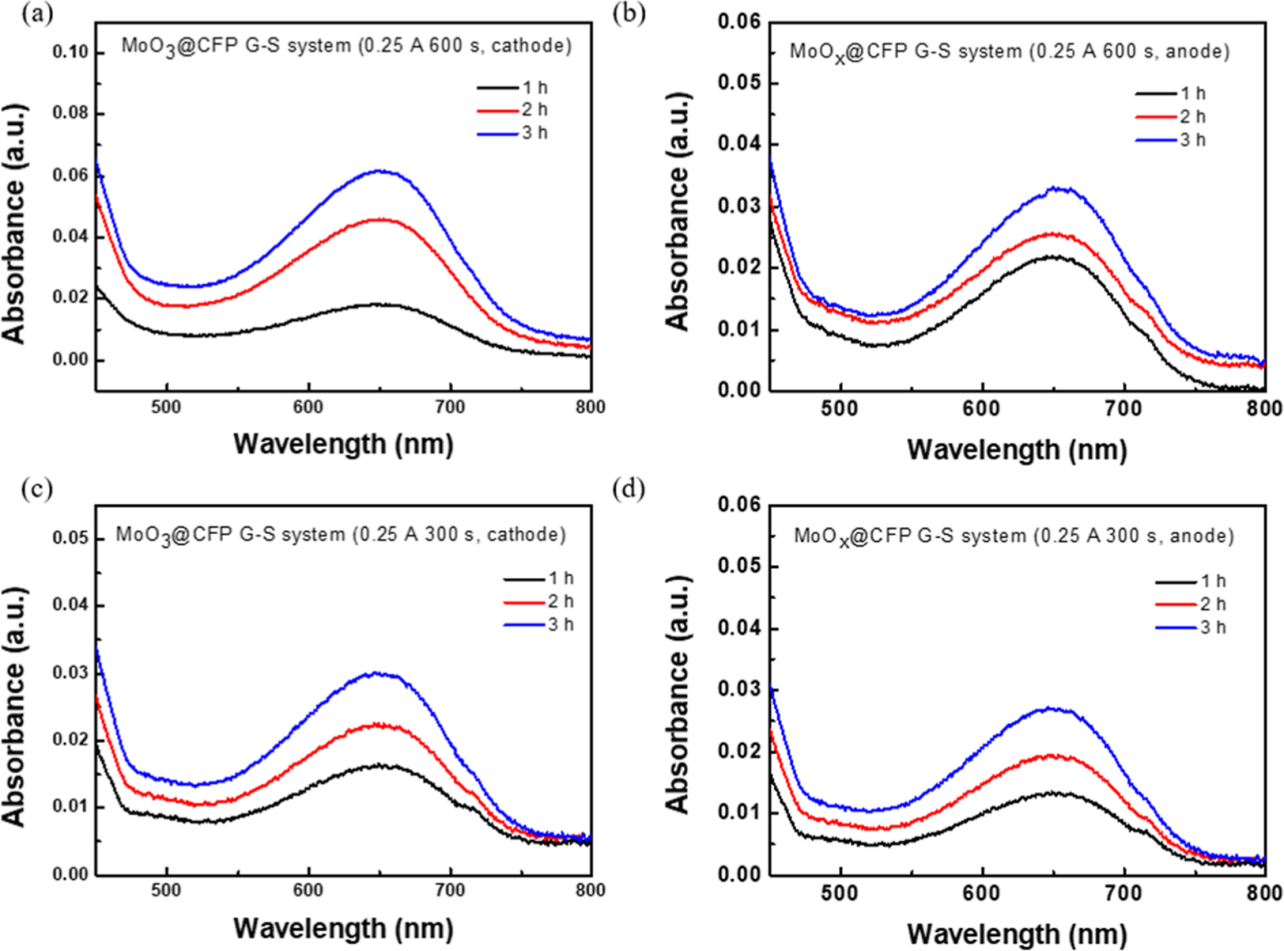
Illustration of the UV–vis absorption spectra of MoO_3_@CFP and MoO_*x*_@CFP in the G–S system for PNRRs under various current densities, reaction times, and operating conditions: (a) cathode configuration, 0.125 A, 600 s; (b) anode configuration, 0.125 A, 600 s; (c) cathode configuration, 0.125 A, 300 s; (d) anode configuration, 0.125 A, 300 s.

**Table 1:** Comparisons of the Performance of MoO_3_@CFP and MoO_*x*_@CFP in PNRR across Solution (S), Gas (G), and G–S System, Including the Ammonia Yield (mg/m^2^), Molar Production Rate (mol·h^–1^), and Concentration-Normalized Rate (mol·g^–1^·h^–1^)

reaction	mg/m^2^	mol·h^–1^	mmol·g ^–1^·h^–1^
MoO_3_@CFP			
solution system	165	1.941 × 10^–6^	2.256
gas system	766	8.995 × 10^–6^	10.455
G–S system	1340	15.901 × 10^–6^	15.144
MoO_*x*_@CFP			
solution system	161	1.892 × 10^–6^	0.701
gas system	591	7.046 × 10^–6^	2.609
G–S system	1021	12.000 × 10^–6^	4.445

### 3.4 PNRR Performances in Different Conditions

PNRR performances of MoO_3_@CFP were systematically evaluated under various experimental conditions, including current density, electrochemical deposition time, magnetic stirring, and the use of sacrificial agents. Figure 5a–d provide a comprehensive overview of how these factors influenced the catalytic efficiency. Figure 5a,b demonstrate that extending the reaction time from 12 to 60 min significantly enhanced UV–vis absorbance at 650 nm corresponding to indophenol blue complex formation, indicating improved ammonia production. This trend suggested that prolonged deposition increased the density and uniformity of active sites on the catalyst surface, thereby sustaining PNRRs. Notably, this effect was amplified at a higher current density (0.25 A), highlighting a synergistic interaction between the current density and deposition duration. As shown in Figure 5c, removing magnetic stirring reduced ammonia production, leading to a lower 650 nm indophenol-blue absorbance compared with the stirred condition. Conversely, as shown in Figure 5c, removing magnetic stirring deteriorated PNRR performance: ammonia production decreased due to reduced mass transfer and reactant diffusion, yielding lower indophenol-blue absorbance at 650 nm compared with the stirred condition. The catalytic performance under different experimental conditions during 1 h PNRR is further analyzed in Figure S3. Extending the deposition time from 300 to 600 s enhanced 650 nm indophenol-blue absorbance, reflecting increased active site density and stability. Shorter deposition times resulted in incomplete activation of active sites, limiting the overall efficiency. Figure 5d compares the conventional practice of adding sacrificial agents to boost PNRR activity and shows that, in our system, introducing a sacrificial agent instead substantially suppresses catalytic performance. The steady growth of absorption signals for the 600 s sample underscores the importance of sufficient preparation time in stabilizing active sites and maintaining reaction consistency. These trends are quantitatively summarized in Table 2. Under optimal conditions (0.25 A, 600 s, and magnetic stirring), the mass-normalized ammonia production rate reached 15.144 mmol·g^–1^·h^–1^. In comparison, the absence of magnetic stirring reduced the production rate to 13.776 mmol·g^–1^·h^–1^, while the addition of sacrificial agents dramatically lowered it to 3.105 mmol·g^–1^·h^–1^. These results emphasize the critical role of optimizing operational parameters to maximize the PNRR efficiency. A steady increase in 650 nm absorbance for the 600 s sample underscores the importance of sufficient preparation time in stabilizing active sites and maintaining reaction consistency. Quantitative results are given in Table 2. Under optimal conditions (0.25 A, 600 s, and magnetic stirring), the mass-normalized ammonia production rate reached 15.144 mmol·g^–1^·h^–1^. In comparison, the absence of magnetic stirring reduced the production rate to 13.776 mmol·g^–1^·h^–1^, while the addition of sacrificial agents dramatically lowered it to 3.105 mmol·g^–1^·h^–1^. These results emphasize the critical role of optimizing operational parameters to maximize the PNRR efficiency. Operationally, the sacrificial agent condition shows a lower 650 nm absorbance (indophenol-blue complex), i.e., reduced NH_3_ signal; we therefore describe it as phenomenological inhibition without invoking site-blocking/adsorption models.

**Figure 5:**
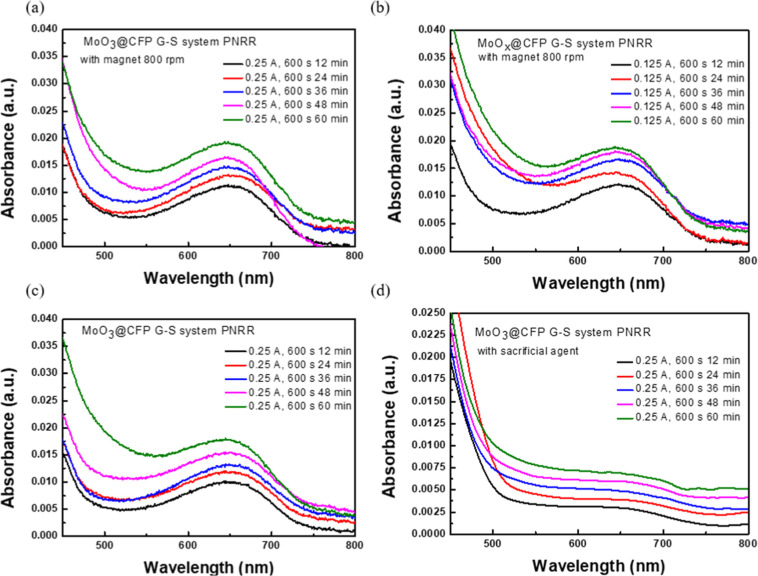
UV–vis absorption spectra of the photocatalytic nitrogen reduction reaction (PNRR) in a G–S system using MoO_3_@CFP and MoO_*x*_@CFP. (a,b) compare the ammonia production over time (12–60 min) at current densities of 0.25 and 0.125 A, respectively. (c) Illustrates the reaction without magnetic stirring, while (d) investigates the effect of introducing a sacrificial agent, revealing a significant suppression of reaction efficiency due to the sacrificial agent.

**Table 2:** Summary of Performance Metrics of MoO_3_@CFP for PNRR under Various Experimental Conditions, Including the Ammonia Yield (mg/m^2^), Molar Production Rate (mol·h^–1^), and Mass-Specific Rate (mmol·g^–1^·h^–1^)

sample	mg/m^2^	mol·h^–1^	mmol·g^–1^·h^–1^
0.25 A, 600 s, with magnet	1105	13.026 × 10^–6^	15.144
0.25 A, 600 s, without magnet	1009	11.848 × 10^–6^	13.776
0.125 A, 600 s, with magnet	1070	12.571 × 10^–6^	14.617
0.25 A, 600 s, sacrificial agent	230	2.707 × 10^–6^	3.105

### 3.5 Photocatalytic Nitrogen Reduction Reaction (PNRR) Performances and Stability

The performance and stability of MoO_3_@CFP in PNRRs were systematically evaluated through multiple reaction cycles and material characterization. Figure 6 demonstrates ammonia production rates of MoO_3_@CFP over five consecutive PNRR cycles with various electrochemical deposition times (12–60 min). The ammonia production efficiency significantly increased with prolonged deposition times, with a peak mass-normalized ammonia production rate of 15.144 mmol·g^–1^·h^–1^ observed at 60 min. The monotonic increase is consistent with changes in film loading and optoelectronic properties under longer deposition, while direct surface-area quantification is not attempted for the CFP-supported thin film. Cycle data are presented as trend demonstrations under identical protocols; no statistical inference is drawn. The structural integrity and compositional stability of the catalyst after repeated PNRR cycles were investigated using material characterization techniques. Figure S4 presents XRD and Raman spectra of MoO_3_@CFP before and after the reaction. XRD patterns (Figure S4a) show that the crystalline structure remained largely unchanged, while the Raman spectra (Figure S4b) indicate no significant shift in the characteristic peaks, confirming that the catalyst maintained its structural framework during prolonged operation. Moreover, XPS analysis (Figure S4c) reveals that the Mo 3d peaks remained at binding energies typical of Mo^6+^, indicating that the oxidation state of molybdenum was preserved throughout the reaction cycles. This further supports the chemical stability of the active Mo centers under PNRR conditions. Further, Figure S5 provides SEM images and EDS mapping of the catalyst surface after PNRR. The SEM image (Figure S5a) shows a stable fibrous structure with no visible degradation, while the elemental mapping (Figure S5b–d) reveals a uniform distribution of Mo and O across the catalyst surface. An elemental analysis confirmed that the weight and atomic percentages of Mo and O remained consistent before and after the reaction, supporting the hypothesis that the catalyst’s active sites were chemically and physically stable under operational conditions. These findings demonstrated the robust performance and structural durability of MoO_3_@CFP, highlighting its potential for sustained use in PNRR applications. Table S2 compares key metrics such as ammonia yield, apparent quantum efficiency (AQE), stability, and reaction conditions. This comparison clearly demonstrated that MoO_3_@CFP achieved one of the highest ammonia production rates under ambient conditions. Its performance surpassed several well-known photocatalysts operating under similar or more-complex conditions, further proving its potential for practical applications in PNRR.

**Figure 6:**
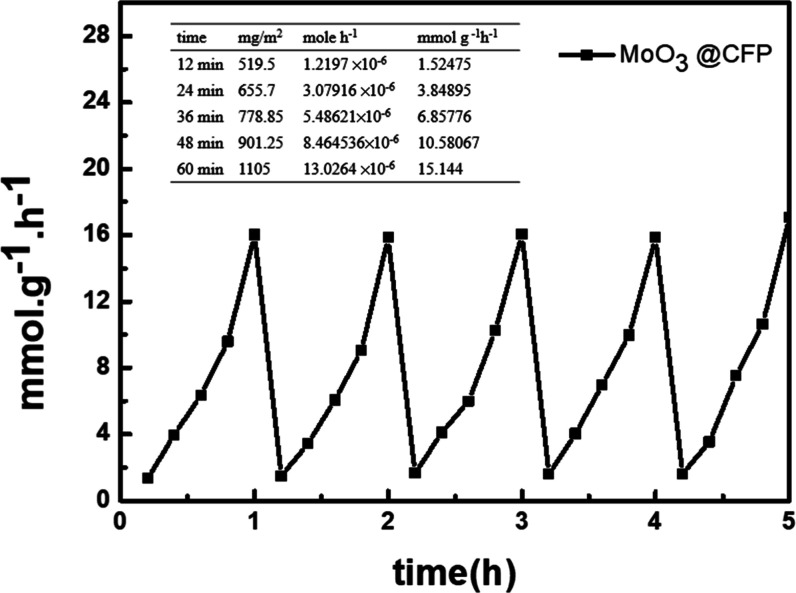
Performance of MoO_3_@CFP in the G–S system (0.25 A, 600 s, with magnetic stirring) for the PNRR. The table presents ammonia production (mg·m^–2^), molar production rate (mol·h^–1^), and mass-specific rate (mmol·g^–1^·h^–1^) at various reaction times (12–60 min). The line chart correlates mass-specific ammonia production rates with reaction times, demonstrating a steady increase in ammonia yields and consistent reproducibility, highlighting the efficiency and stability of this catalytic system.

### 3.6 Mechanism of the Cathode of MoO_3_@CFP

To gain deeper insights into the PNRR mechanism of MoO_3_@CFP, this study systematically analyzed the behavior of photogenerated carriers, charge transfer efficiency, and optical properties. The transient photocurrent response of MoO_3_@CFP (Figure 7a) demonstrated significantly higher intensity and stability under intermittent light illumination compared to pristine CFP, reflecting its efficient generation and separation of photogenerated electron–hole pairs as well as excellent photostability. These results are associated with the unique amorphous structure and abundant surface defects, which create localized energy states that reduce carrier recombinations, as evidenced by the enhanced photocurrent response. The amorphous nature of MoO_3_ introduces disordered structures with numerous active sites, facilitating the rapid migration of photogenerated carriers. Furthermore, the close interaction between MoO_3_ and CFP provides additional pathways for electron transport, reducing recombination and enhancing charge separation. In contrast, the EIS analysis (Figure 7b) revealed a larger charge transfer resistance (*R*_ct_) for MoO_3_@CFP, as indicated by the increased arc radius compared to pristine CFP. This increase may be attributed to the intrinsically lower electrical conductivity of the amorphous MoO_3_ phase, or to the formation of a dense MoO_3_ layer that hindered interfacial electron transport. While this increase in *R*_ct_ suggests a reduction in the charge transfer efficiency at the electrode–electrolyte interface, the overall photocatalytic performance remained promising due to the improved light absorption and photogenerated carrier dynamics. In contrast, Mo=O bonds contribute to nitrogen adsorption and facilitate its initial activation but are limited in their contribution to bulk charge transport due to their localized nature. The combination of these two types of bonds creates a balance between nitrogen adsorption and charge transfer, optimizing the system catalytic performance. The UV–vis absorption spectrum of MoO_3_@CFP (Figure 7c) reveals a substantial enhancement in optical absorption compared to pristine CFP, particularly in the UV and vis regions. This enhancement is likely attributed to defect states, potentially introduced by oxygen vacancies as suggested in prior studies,^57–59^ and synergistic interactions with the carbon fiber substrate. Oxygen vacancies are associated with ligand-to-metal charge transfer (LMCT) and intervalence charge transfer (IVCT) transitions, which facilitate light absorption in the visible spectrum. Additionally, the nanoscale dispersion of MoO_3_ amplifies photon–material interactions, while the carbon fiber substrate enhances interfacial charge separation by providing a highly conductive framework. Synergistic interactions between MoO_3_ and CFP further amplify these effects, improving the overall light-harvesting efficiency. The Tauc plot analysis (Figure 7d) shows that MoO_3_@CFP exhibited a slightly wider bandgap of approximately 1.7 eV compared to 1.4 eV for pristine CFP. This widening of the bandgap may have resulted from structural disorder or quantum confinement effects in the amorphous MoO_3_ phase. Although the bandgap was slightly larger, the enhanced light-harvesting ability and strong absorption in the visible region compensated for the increased excitation threshold. The introduction of defect states within the band structure facilitated additional sub-bandgap transitions, thereby maintaining effective visible light utilization. Taken together, the amorphous structure, surface defects, Mo=O bonds for nitrogen adsorption and activation, and the close interfacial integration with CFP synergistically enhanced the photocatalytic performance of MoO_3_@CFP. Despite the higher *R*_ct_, the improved light absorption, superior photogenerated carrier behavior, and reduced recombination collectively contributed to enhanced nitrogen activation and hydrogenation in the PNRR process, making MoO_3_@CFP a promising candidate for sustainable photocatalytic ammonia synthesis. While amorphous MoO_3_@CFP may introduce trap states, the enhanced TPC together with the visible-region DRS tail and the overall activity indicate that recombination does not dominate under our G–S operation.

**Figure 7:**
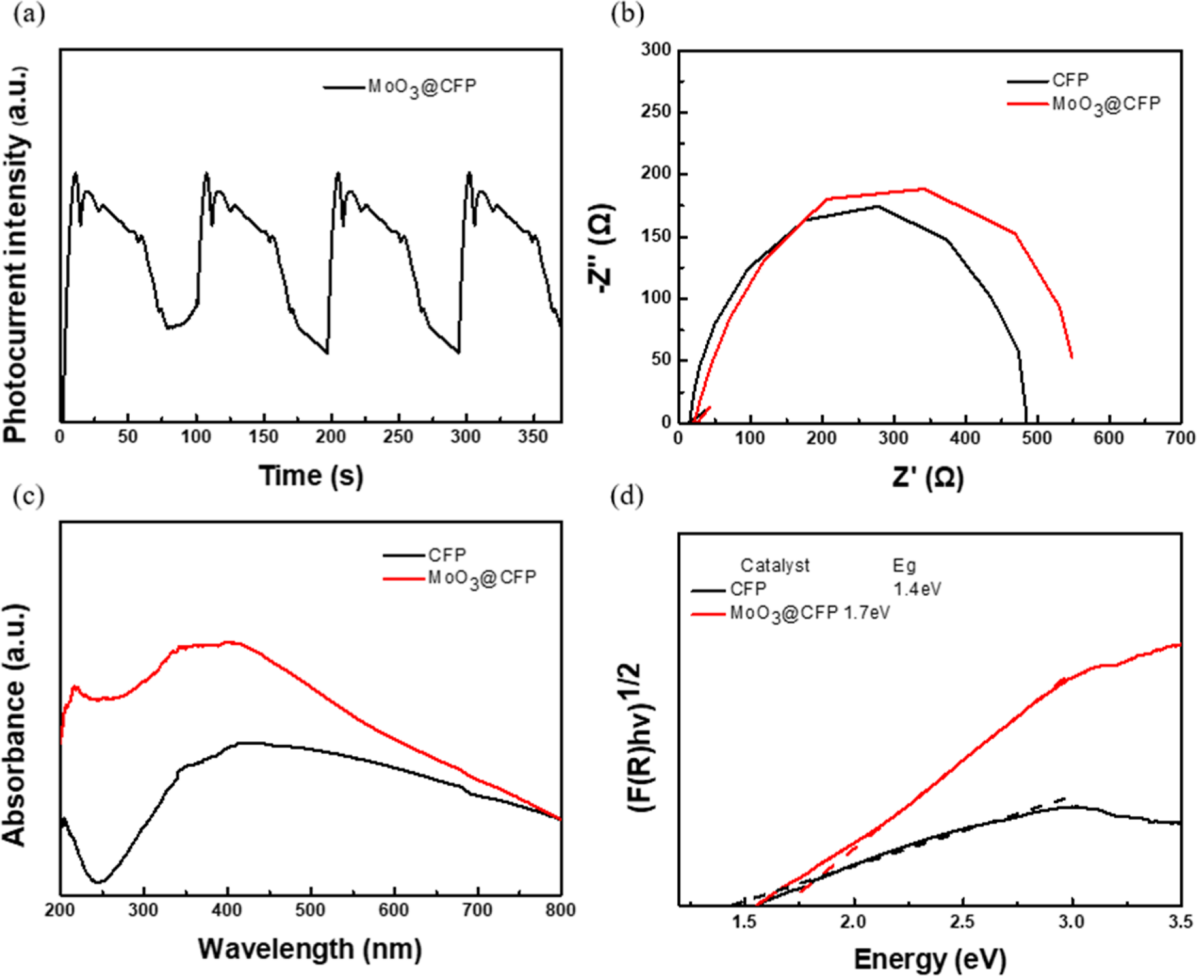
(a) Transient light current response, (b) electrochemical impedance (EIS), (c) UV–vis DRS spectra, (d) and estimated band gaps of MoO_3_@CFP.

## 4 Conclusions

This study highlights the efficiency and practicality of MoO_3_@CFP in PNRRs using a novel G–S system. Under the G–S configuration, MoO_3_@CFP achieves a mass-normalized rate of 15.144 mmol·g^–1^·h^–1^ and maintains performance over five 1 h cycles under ambient-air illumination. The G–S system eliminated the need for external nitrogen gas bubbling, leveraging the natural diffusion of nitrogen from the air. This setup, combined with the use of a low-cost plant lamp as the illumination source, offers a simple and scalable approach for PNRRs. The plant lamp’s spectrum effectively excited the MoO_3_@CFP catalyst, demonstrating comparable or superior efficiency to traditional systems. The catalyst preparation process was straightforward and cost-effective, with the amorphous MoO_3_@CFP structure providing abundant active sites and surface defects critical for nitrogen activation. The G–S system overcame the limitations of single-phase systems by integrating efficient mass transfer and nitrogen availability, achieving stable and high ammonia production rates. This unique combination of features not only enhanced the catalytic performance but also represents a significant step toward sustainable nitrogen reduction technologies. By introducing the G–S system, this study opens a new pathway for developing practical, environmentally friendly, and scalable photocatalytic systems, forging new possibilities for energy-efficient ammonia production and contributing to global sustainability efforts.

## Supplementary Material



## Data Availability

All data is available throughout the manuscript and Supporting Information files.

## Notes

The schematic of the experimental setup; UV–vis absorption spectra of MoO_3_@CFP in a G–S system for PNRR after 1 h under varying operating conditions; UV–vis absorption spectra MoO_*x*_@CFP for the solution system, gas system, and G–S system in photocatalytic PNRR at different reaction times; XRD patterns, Raman spectra, and XPS of MoO_3_@CFP before and after reaction; surface morphology and elemental distribution of MoO_3_@CFP after 60 min of PNRR; the weights of samples of cathode and anode; the comparison of NRR performance in various photo reaction systems; reactor and operating parameters (PDF)
