# Supplementary material for: Defect-Rich Gas–Solution Photocatalytic Systems for Nitrogen Reduction Reactions: Enabling Energy and Carbon Reductions
Source: ACS Omega. 2025 Nov 25;10(48):58821–31. doi: 10.1021/acsomega.5c07318 (PMC12771258; doi:10.1021/acsomega.5c07318)
Supplement: Supplementary file 1 [file ao5c07318_si_001.pdf]

Supporting information

Defect-Rich Gas-Solution Photocatalytic Systems for Nitrogen Reduction Reactions: Enabling Energy and Carbon Reductions

Shih-Mao Peng[1], Muhammad Saukani[2], Jen-Chang Yang[3,4], Tsung-Rong Kuo[3,4,*]

[1] Graduate Institute of Biomedical Materials & Tissue Engineering, Taipei Medical University, New Taipei City 23564, Taiwan

[2] Department of Mechanical Engineering, Faculty of Engineering, Universitas Islam Kalimantan MAB, Banjarmasin 70124, Kalimantan Selatan, Indonesia

[3] Graduate Institute of Nanomedicine and Medical Engineering, College of Biomedical Engineering, Taipei Medical University, Taipei 23564, Taiwan

[4] International Ph.D. Program in Biomedical Engineering, College of Biomedical Engineering, Taipei Medical University, New Taipei City 23564, Taiwan

* Corresponding author: trkuo@tmu.edu.tw

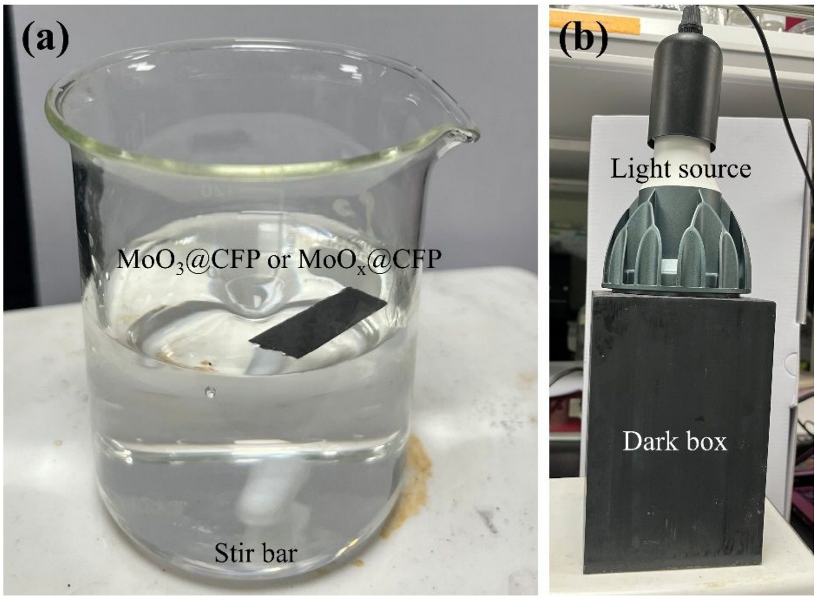

Figure S1. The schematic of the experimental setup: (a) The MoO_3@CFP or MoO_x@CFP was floated at the liquid-air interface. The beaker was then placed inside the dark box. (b) All experiments were conducted inside a dark box, a light-tight enclosure with top-down illumination serving as the photocatalytic light source.

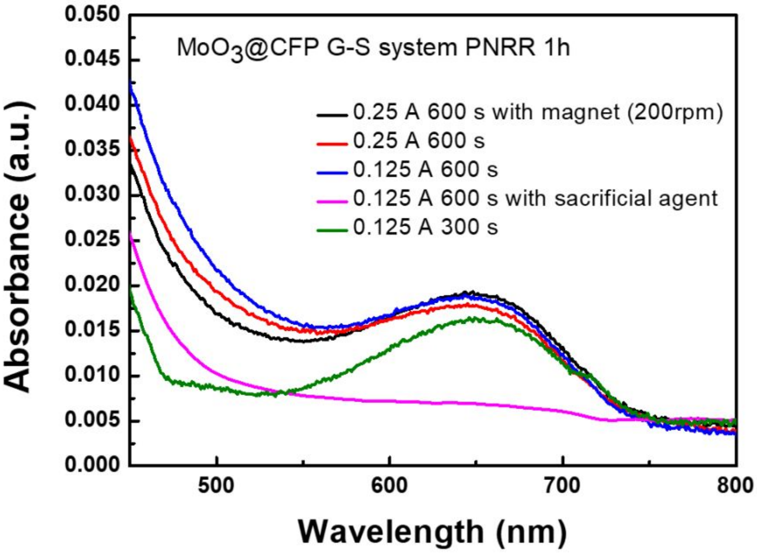

Figure S2. Illustration of the UV-Vis absorption spectra of MoO_3@CFP in a G-S system for PNRR after 1 hour under varying operating conditions. The comparison includes current density (0.25 A vs. 0.125 A), the presence of magnetic stirring, the use of a sacrificial agent, and charging duration (600 s vs. 300 s).

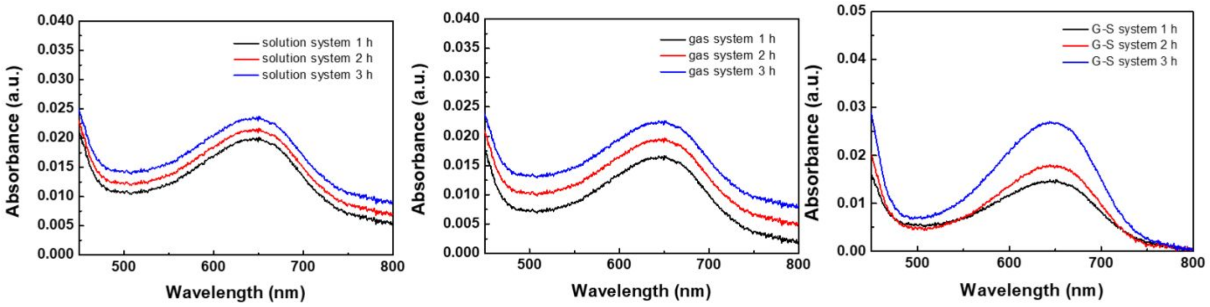

Figure S3. MoO_x@CFP presents the UV-Vis absorption spectra for the solution system (a), gas system (b), and G-S system (c) in photocatalytic PNRR at different reaction times (1 h, 2 h, 3 h).

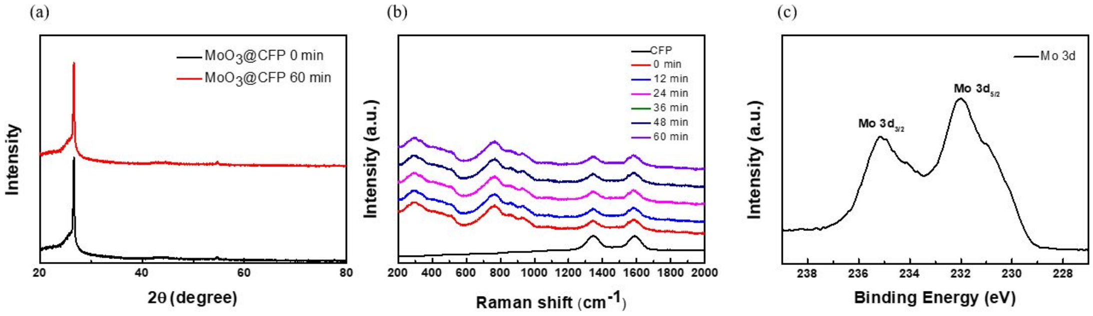

Figure S4. (a) XRD patterns, (b) Raman spectra, and (c) XPS of MoO_3@CFP before and after reaction (1 h).

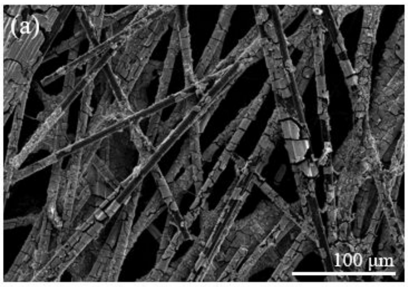

Element	Weight (%)	Atomic (%)
C	43.40	61.69
Mo	24.84	33.89
O	31.84	33.89

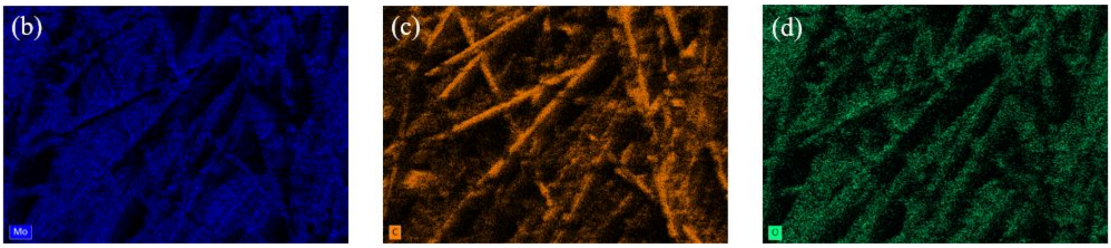

Figure S5. Surface morphology and elemental distribution of MoO_3@CFP after 60 min of PNRR: (a) SEM overview; (b) higher-magnification SEM; (c) EDX elemental maps (C, Mo, O) along the CFP fibers; (d) inset table, area-averaged EDX compositions from multiple non-overlapping fields (semi-quantitative on CFP).

Table S1. The weights of five samples were measured prior to and following electrochemical deposition, and the average weights of cathode and anode were determined.

**Sample**	**CFP (mg)**	**MoO_3@CFP (mg)**	**CFP (mg)**	**MoO_x@CFP (mg)**
Sample 1	16.6	17.4	18.8	21.8
Sample 2	15.7	16.3	18.0	20.4
Sample 3	16.0	16.9	18.6	21.8
Sample 4	15.6	16.6	16.5	20.0
Sample 5	17.3	18.3	17.0	18.4
**Average**	-	**0.86**	-	**2.7**

Table S2. The comparison of NRR performance in various photo reaction systems.

Photocatalyst	Illumination	Quantification method	Reaction medium	NH_3 yield; AQE[a]/AQY[n] (%)	Stability	Ref.
MoO_3 @CFP	24 W plant lamp (range: 380 nm to 780 nm)	Indophenol blue	Water; 25 °C	15.144 mmol·g^{-1}·h^{-1}	5 cycles; 1 h	This work
1T MoS_2/Cds	720 nm ≥ λ ≥ 400 nm; (250 W Na)	Nessler's reagent	Water/ methanol; RT	8220.83 μmol·L^{-1}·h^{-1}·g^{-1}; AQE: 4.424	3 cycles	[1]
B-C_3N_4 (MoO_2)	λ ≥ 400 nm; (300 W Xe)	Nessler's reagent	Water/ methanol; RT	1.68 mmol·g^{-1}·h^{-1}; AQE: 0.62 (400 nm)	4 cycles	[2]
$Bi_2O_2CO_3$ nanosheet	λ ≥ 420 nm; (300 W Xe)	Nessler's reagent and ion chromatograph	Water/ Na_2SO_3; RT	1,178 μmol·L^{-1}·g^{-1}·h^{-1}; AQE: 0.07 (420 nm)	4 cycles	[3]
Bi_5O_7Br nanostructure	λ ≥ 400 nm; (300 W Xe)	Nessler's reagent	Water; 25 °C	12.72 mmol·g^{-1}·h^{-1}	No cycle	[4]
Bi_2MoO_6 sphere	λ ≥ 420 nm; (300 W Xe)	Nessler's reagent	Water; RT	1.3 mmol·g^{-1}·h^{-1}; AQE: 0.73 (500 nm)	13 cycles	[5]
Bi_5O_7Br nanotube	λ ≥ 400 nm; (300 W Xe)	Nessler's reagent	Water; 25 °C	1.38 mmol·g^{-1}·h^{-1}; AQE: 2.3 (420 nm)	4 cycles; 10 h	[6]
Fe-BiOCl nanosheet	Full-spectrum; (300 W Xe)	Indophenol blue	Water; RT	1.022 mmol·g^{-1}·h^{-1}; AQE: 1.8 (420 nm)	5 cycles	[7]
NC-g-C_3N_4	(300 W Xe, AM 1.5G, 100 mW·cm^{-2})	Nessler's reagent	Water/ methanol; RT	1.59 mmol·g^{-1}·h^{-1}	No	[8]
B-g-C_3N_4	λ ≥ 420 nm; (300 W Xe)	Nessler's reagent	Water/ Na_2SO_3; 25 °C	435.28 μmol·L^{-1}·g^{-1}·h^{-1}	5 cycles	[9]
Ru-CoS/ g-C_3N_4	λ ≥ 420 nm; (300 W Xe, 200 mW·cm^{-2})	Indophenol blue	Water/ methanol; RT	0.438 mmol·g^{-1}·h^{-1}; AQE: 1.28 (400 nm)	12 cycles	[10]

Ti_3C_2Tx/TiO_2	spectrum; (Xe, 250 mW·cm^{-2}) λ = 630 nm (25 mW·cm^{-2})	Nessler's reagent and ion chromatograph	Water; 25 °C	422 μmol·L^{-1}·g^{-1}·h^{-1} (full-spectrum); AQE: 0.05 (630 nm);	10 cycles	[11]
B-g-C_3N_4 nanosheet	λ ≥ 400 nm; (250 W Xe, 0.5 W·cm^{-2})	Nessler's reagent	Water/ Na_2SO_3; RT	313.9 μmol·L^{-1}·g^{-1}·h^{-1}	5 cycles	[12]
Au/g-C_3N_4 hollow sphere	λ ≥ 420 nm; (300 W Xe)	Nessler's reagent	Water/ methanol; RT	357.3 μmol·L^{-1}·g_{cat}^{-1}·h^{-1}; AQY: 0.64 (550 nm)	4 cycles	[13]
Fe-BiOBr nanosheet	λ ≥ 420 nm; (300 W Xe)	Nessler's reagent	Water; 25 °C	382.68 μmol·L^{-1}·g_{cat}^{-1}·h^{-1}	4 cycles	[14]

RT: room temperature; No: No cycle

Table S3. Reactor and operating parameters.

Block	Field	Value / Method
Gas	Source	Ambient air (non-bubbling, diffusion-controlled)
	N_2 purity	Ambient air
	Flow (sccm)	Sealed, no flow
	Humidity	RH = 75%
	Leak test	n/a
Liquid	Electrolyte	Ultrapure water (100 mL)
	pH	pH = 7
	Stirring	On/off per test
Catalyst / Electrode	Material	MoO_3@CFP
	Active area	2 cm² (2 × 1 cm²)
	Loading	0.86 mg total ⇒ 0.43 mg·cm^{-2}
	Gas-facing vs solution-facing	Top face sees gas/light; bottom face contacts solution
Light	Source	24-W plant lamp; 380-780 nm; peaks at ~452 and ~567 nm; CRI ≈ 97; CCT ≈ 5120 K
	Distance	≈ 14 cm (lamp to catalyst)
	Intensity at catalyst plane	n/a
	PPFD	150 µmol·m^{-2}·s^{-1} @100 cm (for reference only)
Temperature / Time	Temperature	Room temperature
	Run time	12-60 min per cycle; cycling 5× (1 h each)
	Humidity	RH = 75%

Reference

1. Sun, B.; Liang, Z.; Qian, Y.; Xu, X.; Han, Y.; Tian, J., Sulfur Vacancy-Rich O-Doped 1T-MoS(2) Nanosheets for Exceptional Photocatalytic Nitrogen Fixation over CdS. *ACS Appl Mater Interfaces* **2020,** *12*, 7257-7269.
2. Ran, Y.; Yu, X.; Liu, J.; Cui, J.; Wang, J.; Wang, L.; Zhang, Y.; Xiang, X.; Ye, J., Polymeric carbon nitride with frustrated Lewis pair sites for enhanced photofixation of nitrogen. *Journal of Materials Chemistry A* **2020,** *8*, 13292-13298.
3. Feng, Y.; Zhang, Z.; Zhao, K.; Lin, S.; Li, H.; Gao, X., Photocatalytic nitrogen fixation: Oxygen vacancy modified novel micro-nanosheet structure Bi(2)O(2)CO(3) with band gap engineering. *J Colloid Interface Sci* **2021,** *583*, 499-509.
4. Li, P.; Zhou, Z.; Wang, Q.; Guo, M.; Chen, S.; Low, J.; Long, R.; Liu, W.; Ding, P.; Wu, Y.; Xiong, Y., Visible-Light-Driven Nitrogen Fixation Catalyzed by Bi(5)O(7)Br Nanostructures: Enhanced Performance by Oxygen Vacancies. *J Am Chem Soc* **2020,** *142*, 12430-12439.
5. Hao, Y.; Dong, X.; Zhai, S.; Ma, H.; Wang, X.; Zhang, X., Hydrogenated Bismuth Molybdate Nanoframe for Efficient Sunlight-Driven Nitrogen Fixation from Air. *Chemistry* **2016,** *22*, 18722-18728.
6. Wang, S.; Hai, X.; Ding, X.; Chang, K.; Xiang, Y.; Meng, X.; Yang, Z.; Chen, H.; Ye, J., Light-Switchable Oxygen Vacancies in Ultrafine Bi(5) O(7) Br Nanotubes for Boosting Solar-Driven Nitrogen Fixation in Pure Water. *Adv Mater* **2017,** *29*.
7. Zhang, N.; Li, L.; Shao, Q.; Zhu, T.; Huang, X.; Xiao, X., Fe-Doped BiOCl Nanosheets with Light-Switchable Oxygen Vacancies for Photocatalytic Nitrogen Fixation. *ACS Applied Energy Materials* **2019,** *2*, 8394-8398.

8. Xue, Y.; Guo, Y.; Liang, Z.; Cui, H.; Tian, J., Porous g-C(3)N(4) with nitrogen defects and cyano groups for excellent photocatalytic nitrogen fixation without co-catalysts. *J Colloid Interface Sci* **2019,** *556*, 206-213.
9. Liang, C.; Niu, H.-Y.; Guo, H.; Niu, C.-G.; Huang, D.-W.; Yang, Y.-Y.; Liu, H.-Y.; Shao, B.-B.; Feng, H.-P., Insight into photocatalytic nitrogen fixation on graphitic carbon nitride: Defect-dopant strategy of nitrogen defect and boron dopant. *Chemical Engineering Journal* **2020,** *396*.
10. Yuan, J.; Yi, X.; Tang, Y.; Liu, M.; Liu, C., Efficient Photocatalytic Nitrogen Fixation: Enhanced Polarization, Activation, and Cleavage by Asymmetrical Electron Donation to N□N Bond. *Advanced Functional Materials* **2019,** *30*.
11. Hou, T.; Li, Q.; Zhang, Y.; Zhu, W.; Yu, K.; Wang, S.; Xu, Q.; Liang, S.; Wang, L., Near-infrared light-driven photofixation of nitrogen over Ti3C2Tx/TiO2 hybrid structures with superior activity and stability. *Applied Catalysis B: Environmental* **2020,** *273*.
12. Wang, W.; Zhou, H.; Liu, Y.; Zhang, S.; Zhang, Y.; Wang, G.; Zhang, H.; Zhao, H., Formation of B□N□C Coordination to Stabilize the Exposed Active Nitrogen Atoms in g-C(3) N(4) for Dramatically Enhanced Photocatalytic Ammonia Synthesis Performance. *Small* **2020,** *16*, e1906880.
13. Guo, Y.; Yang, J.; Wu, D.; Bai, H.; Yang, Z.; Wang, J.; Yang, B., Au nanoparticle-embedded, nitrogen-deficient hollow mesoporous carbon nitride spheres for nitrogen photofixation. *Journal of Materials Chemistry A* **2020,** *8*, 16218-16231.
14. Liu, Y.; Hu, Z.; Yu, J. C., Fe Enhanced Visible-Light-Driven Nitrogen Fixation on BiOBr Nanosheets. *Chemistry of Materials* **2020,** *32*, 1488-1494.
